# Novel Mechanisms and Therapeutic Targets for Ischemic Stroke: A Focus on Gut Microbiota

**DOI:** 10.3389/fncel.2022.871720

**Published:** 2022-05-17

**Authors:** Zeyu Bao, Zeyu Zhang, Guoyang Zhou, Anke Zhang, Anwen Shao, Feng Zhou

**Affiliations:** Department of Neurosurgery, The Second Affiliated Hospital, School of Medicine, Zhejiang University, Hangzhou, China

**Keywords:** ischemic stroke, gut microbiota, microbiome, mechanism, target, treatment

## Abstract

Ischemic stroke is the most common type of stroke with limited treatment options. Although the pathological mechanisms and potential therapeutic targets of ischemic stroke have been comprehensively studied, no effective therapies were translated into clinical practice. Gut microbiota is a complex and diverse dynamic metabolic ecological balance network in the body, including a large number of bacteria, archaea, and eukaryotes. The composition, quantity and distribution in gut microbiota are found to be associated with the pathogenesis of many diseases, such as individual immune abnormalities, metabolic disorders, and neurodegeneration. New insight suggests that ischemic stroke may lead to changes in the gut microbiota and the alterations of gut microbiota may determine stroke outcomes in turn. The link between gut microbiota and stroke is expected to provide new perspectives for ischemic stroke treatment. In this review, we discuss the gut microbiota alterations during ischemic stroke and gut microbiota-related stroke pathophysiology and complications. Finally, we highlight the role of the gut microbiota as a potential therapeutic target for ischemic stroke and summarize the microbiome-based treatment options that can improve the recovery of stroke patients.

## Introduction

Ischemic stroke accounts for about 70–80% of all stroke patients (Feigin et al., 2003). The main cause of ischemic stroke is insufficient blood and oxygen supply to the brain. Embolus or thrombus forms could block cerebrovascular, which make blood supply of local brain tissue decrease, thereby causing brain tissue damage (Dirnagl et al., 1999). Currently, there are two main therapies for ischemic stroke: thrombolysis and thrombectomy. However, their applications in clinical practice are still very limited due to the short treatment window (Fisher and Saver, 2015). In recent years, the pathological mechanisms and potential therapeutic targets of ischemic stroke have been comprehensively studied, including excitotoxicity, oxidative stress, neuroinflammation, apoptosis, and blood-brain barrier (BBB) disruption (Zhang Z. Y. et al., 2019). However, no effective therapies were translated into clinical practice. Therefore, it still needs our great attention to find new therapies to prevent or reduce neuronal injury after ischemic stroke.

Gut microbiota is a complex and diverse dynamic metabolic ecological balance network, including a large number of bacteria, archaea, and eukaryotes. Gut microbiota is formed at birth and retains maternal characteristics. After exposure to a complex microbiome, babies develop a largely stable gut microbiota by the time they are 1–3 years old (Mackie et al., 1999; Palmer et al., 2007). But it can also change due to the host’s dietary habits, stress, antibiotic use, and aging (Claesson et al., 2011; Wu and Hui, 2011; Shimizu, 2018). The composition, quantity and distribution in gut microbiota are associated with the pathogenesis of a wide variety of diseases, such as individual immune abnormalities, metabolic disorders, and neurodegeneration (Duvallet et al., 2017). At present, biphase associations between gut microbiota and many body organs have been identified, including gut-cardiac axis, gut-thyroid axis, and gut-liver axis (Koszewicz et al., 2021). The brain and gut microbiota can interact with each other not only through neuronal pathways but also through microbial metabolites, hormones, and the immune system, termed the gut-brain-microbiota axis (GBMAx) (El Aidy et al., 2015; Durgan et al., 2019). Ischemic stroke may lead to changes in the gut microbiota, which can affect surrounding or distant tissues and organs, causing serious damages to liver, kidney, lung, gastrointestinal tract, cardiovascular system, and so on. In turn, changes in the gut microbiota may be one of the risk factors for ischemic stroke and determine stroke outcomes (de Jong et al., 2016). The link between gut microbiota and stroke is expected to provide new perspectives for ischemic stroke treatment.

This article reviews the gut microbiota alterations during ischemic stroke, gut microbiota-related stroke pathophysiology and complications, as well as potential therapeutic strategies targeting gut microbiota for ischemic stroke.

### Alterations of Gut Microbiota During Ischemic Stroke

Multiple clinical and animal studies have revealed the changes in gut microbiota following ischemic stroke. One case-control study showed that the gut microbiota was significantly disrupted in patients with ischemic stroke and transient ischemic attack compared to controls. The main manifestations were the increase of opportunistic pathogens and the decrease of commensal or beneficial genera (Yin et al., 2015). In another study, the gut microbiota of ischemic stroke patients had more short chain fatty acids producer compared to healthy controls. In addition, it was found that the genus Enterobacter was significantly correlated with good outcomes (Li et al., 2019). An animal experiment based on the mouse middle cerebral artery occlusion (MCAO) model showed that ischemic stroke resulted in reduced species diversity and bacterial overgrowth of Bacteroidetes in the gut (Singh et al., 2016). Another study found that the levels of Bacteroidetes phylum and Prevotella genus were significantly increased in the gut of cynomolgus monkeys after MCAO, while Firmicutes phylum as well as Faecalibacterium, Oscillospira, and Lactobacillus genera were decreased, Oscillobacter, and Lactobacillus were decreased. In addition, intestinal mucosal damage was also observed (Chen et al., 2019c).

In addition to causing gut microbiota dysbiosis, ischemic stroke may also facilitate the translocation and dissemination of selective strains of bacteria that originated from the host gut microbiota. Infection is usually more likely to be observed after an ischemic stroke. Stanley et al. (2016) demonstrated that the microbial community in the lungs of post-stroke mice were derived from the small intestine of the host using high-throughput 16S rRNA gene amplicon sequencing and bioinformatics analyses.

Changes in gut bacteria can also be a factor in ischemic stroke. Significant microbiological disorders have been detected in inflammatory bowel disease (including Crohn’s disease and ulcerative colitis) and chronic kidney disease, all of which were found to be risk factors for ischemic stroke (Lee et al., 2010; Kristensen et al., 2014; Xiao et al., 2015). In addition, the composition of gut bacteria of people at high risk of stroke is also different from that of the normal population. Compared with the low-risk group of stroke, the levels of opportunistic pathogens among the people of high-risk group were found to be higher, and the difference of enterobacteriaceae was the most obvious. The people of low-risk group had higher concentration of butyrate-producing bacteria, such as Lachnospiraceae and Ruminococcaceae (Zeng et al., 2019). These findings may imply that disruption of microbial homeostasis in gut may precede the development of stroke. Therefore, it is feasible to predict and prevent stroke in advance by observing changes in intestinal flora.

There are also differences in gut microbiota among stroke patients of different ages. The incidence of stroke is closely related to age, with about 70–80% of ischemic strokes occurring in people over 65 years of age (Ovbiagele and Nguyen-Huynh, 2011), and age plays an important role in the development and prognosis of stroke (Yager et al., 2006; Manwani et al., 2011). Some pathophysiological processes are associated with aging, such as chronic inflammation and decreased immune function, can affect functional recovery after stroke and lead to poor prognosis in the elderly (Crapser et al., 2016; Ritzel et al., 2018). And On the other hand, the composition of gut microbes can be influenced by environment, disease and eating habits, as well as age and gender differences (Coman and Vodnar, 2020). The composition of the gut microbiota changes and the diversity diminishes as we get older. When the gut microbiome disorders, it has a detrimental effect on normal physiological activity and is also thought to affect age-related neurodegenerative diseases such as Alzheimer’s disease, Parkinson’s disease and Huntington’s disease (Mulak and Bonaz, 2015; Wasser et al., 2020; Escobar et al., 2022). Studies have shown that age plays an important role in the interaction between gut microbiota and stroke. Bacteroidetes and Firmicutes dominated the gut microbiota of both young and old adults. In older adults, the relative abundance of Firmicutes increased, and the content of SCFAs-producing bacteria and butyrate level decreased significantly (Biagi et al., 2010; Claesson et al., 2011), and intestinal permeability of the elderly was significantly higher than that of the young (Lee et al., 2020), which makes the older more susceptible to inflammatory response. And according to another study in mice, stroke outcomes can be improved in older mice by transplanting microbiota from younger mice. In contrast, after acquiring the microbiome of the older mice, the younger mice increased functional impairment after stroke (Spychala et al., 2018). In addition, age is an independent risk factor for post-stroke infection, the frequency and severity of infection after stroke were higher in the elderly. This may be related to the impaired integrity of the intestinal barrier, the entry of intestinal bacteria into peripheral tissues through the damaged barrier. And another possible explanation is intestinal inflammation. Higher levels of pro-inflammatory cytokines were detected in older patients than in younger patients (Crapser et al., 2016; Spychala et al., 2018; Blasco et al., 2020).

In addition, differences in the performance of gut microbiota after stroke also exist between genders. As two common intestinal bacteria, Bacteroidetes and Firmicutes, there are more Firmicutes detected in the males’ gut when they had a BMI of less than 33 compared with females. And when BMI is greater than 33, males have an advantage over females in the abundance of Bacteroidetes. In addition, the abundance of Lactobacilli in female is much higher than that in male (Haro et al., 2016). And there are also gender differences in post-stroke outcomes. In some studies, adult females have better recovery outcomes than males after stroke (Toung et al., 1998; Branyan and Sohrabji, 2020). And in middle age (45–55 years), male stroke patients have a higher mortality rate than female stroke patients (Redon et al., 2011). The prognosis of senile stroke women is proved significantly worse. This suggests that estrogen may play a protective role in the development of stroke. In addition, there were gender differences in the expression of bacterial metabolites after stroke. Fecal butyrate levels in male were significantly lower than in female after stroke (Ahnstedt et al., 2020), but LPS was found to be higher in male. After induced stroke, the male mouse model had greater intestinal permeability (Ahnstedt et al., 2020; El-Hakim et al., 2021). This suggests that male patients are more susceptible to intestinal microbiota translocation and post-stroke infection after stroke. There were also differences between male and female in inflammatory responses after stroke. Females expressed more Treg cells, while males had higher concentrations of CD8 + T cells (Jackson et al., 2019; Ahnstedt et al., 2020; Blasco et al., 2020). However, no more studies have clearly proved that gender can cause changes in the composition of intestinal microbiota in stroke patients, so the association between gender and stroke and intestinal microbiota needs further exploration.

### Pathophysiological Mechanisms of the Interaction Between Gut Microbiota and Ischemic Stroke

**FIGURE 1:**
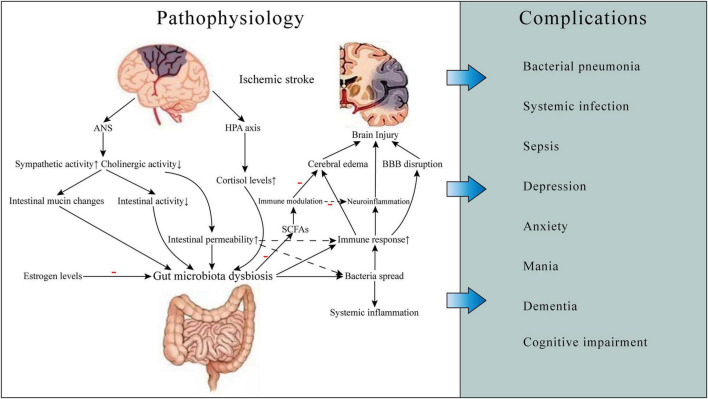
Gut microbiota-related ischemic stroke pathophysiology and complications. Ischemic stroke can cause gut microbiota dysbiosis, which may result in increased gut permeability and worsening brain injury, thereby leading to some complications such as infections and neuropsychiatric disorders and poor prognosis. The mechanisms involved include neuroendocrine pathways, bacterial metabolite, and immune response. ANS, autonomic nervous system; HPA, hypothalamic-pituitary-adrenal; BBB, blood-brain barrier; SCFAs, short-chain fatty acids.

The interaction between gut microbiota and ischemic stroke plays an important role in the occurrence, development and outcomes of stroke. We summarize the relevant pathophysiological mechanisms, including neuroendocrine pathways, bacterial metabolite, and immune response (Figure 1). The studies exploring this interaction and relevant mechanisms are listed in Table 1.

**TABLE 1:** Related studies exploring the relationship between gut microbiota and ischemic stroke.

Researchers and years	Studied species	Related study results	Findings
Caso et al., 2009	Fischer rats	Stress following ischemic stroke resulted in decreased intestinal activity, increased intestinal permeability, translocation of intestinal bacteria, and increased expression of intestinal inflammatory enzymes such as COX-2 and iNOS.	Ischemic stroke can cause increased intestinal permeability and bacterial dispersal through sympathetic activation.
Park et al., 2020	SD rats	Reproductive senescent females had significant gut dysbiosis at baseline and after ischemic stroke compared with adult females. Gut metabolites were differently affected by estrogen treatment in reproductive senescent females and adult females.	Microbial gut can be altered by reproductive senescence in female rats at baseline and after ischemic stroke and estrogen may impact stroke recovery differently in adult and reproductive senescent females due to an age-specific effect on gut microbiota and metabolites.
Chen et al., 2019b	SD rats	Oral administration of non-absorbable antibiotics reduced neurological impairment and the cerebral infarct volume, relieved cerebral edemas, and decreased blood lipid levels by altering the gut microbiota. Ischemic stroke decreased intestinal levels of SCFAs. Transplanting fecal microbiota rich in these metabolites was an effective means of treating the condition.	Interfering with the gut microbiota by transplanting fecal bacteria rich in SCFAs and supplementing with butyric acid were found to be effective treatments for cerebral ischemic stroke.
Chen et al., 2019a	SD rats	The combination of Puerariae Lobatae Radix and Chuanxiong Rhizoma protected the brain-gut barriers by increasing claudin-5 and ZO-1 levels, weakened the gut microbiota translocation by decreasing diamine oxidase, lipopolysaccharide and d-lactate, and effectively improved the neurological function after ischemic stroke.	Ischemic stroke can cause gut microbiota dysbiosis, increase intestinal permeability, disrupte the gut barrier and triggere gut microbiota translocation. The combination of Puerariae Lobatae Radix and Chuanxiong Rhizoma can reduce post-stroke brain damage through relieving the gut microbiota dysbiosis and brain-gut barriers disruption.
Chen et al., 2019c	Cynomolgus monkeys	The levels of the Bacteroidetes phylum and Prevotella genus were significantly increased, while the Firmicutes phylum as well as the Faecalibacterium, Oscillospira, and Lactobacillus genera were decreased after cerebral infarction. Gut-originating SCFAs were significantly decreased 6 and 12 months after cerebral infarction. The increases in plasma LPS, TNF-α, IFN-γ, and IL-6 after cerebral infarction coincided with overgrowth of the Bacteroidetes phylum.	Cerebral infarction induces persistent host gut microbiota dysbiosis, intestinal mucosal damage, and chronic systemic inflammation.
Stanley et al., 2018	C57BL/6 mice	Ischemic stroke induced changes in the gut microbiota in mice, including an increased abundance of Akkermansia muciniphila and an excessive abundance of clostridial species.	Ischemic stroke can induce far-reaching and robust changes to the intestinal mucosal microbiota.
Lee et al., 2020	C57BL/6 mice	Young fecal transplants contained much higher SCFAs levels and related bacterial strains. Aged stroke mice receiving young fecal transplant gavage had less behavioral impairment, and reduced brain and gut inflammation. SCFAs-producers supplement alleviated post-stroke neurological deficits and inflammation, and elevated gut, brain and plasma SCFAs concentrations in aged stroke mice.	The poor stroke recovery in aged mice can be reversed *via* post-stroke bacteriotherapy following the replenishment of youthful gut microbiome *via* modulation of immunologic, microbial, and metabolomic profiles in the host.
Patnala et al., 2017	C57BL/6 mice	Sodium butyrate mediated neuroprotection after ischemic stroke by epigenetically regulating the microglial inflammatory response, *via* downregulating the expression of pro-inflammatory mediators, TNF-α and NOS2, and upregulating the expression of anti-inflammatory mediator IL10, in activated microglia.	Sodium butyrate can epigenetically modify microglial behavior from pro-inflammatory to anti-inflammatory which could mitigate microglia-mediated neuroinflammation after ischemic stroke.
Schulte-Herbruggen et al., 2009	C57BL/6 mice	Peyer’s patches in gut revealed a significant reduction of T and B cell counts after cerebral ischemia, while no differences in natural killer cells and macrophages were observed.	Cerebral ischemia may cause changes in intestinal immune cell populations.
Benakis et al., 2016	C57BL/6 mice	Antibiotic-induced alterations in the intestinal flora reduced ischemic brain injury in mice, an effect transmissible by fecal transplants. Intestinal dysbiosis altered immune homeostasis in the small intestine, leading to an increase in regulatory T cells and a reduction in interleukin (IL)-17-positive γδ T cells through altered dendritic cell activity. Dysbiosis suppressed trafficking of effector T cells from the gut to the leptomeninges after stroke.	Gut commensal microbiota may affect ischemic stroke outcome by regulating intestinal γδ T cells.
Sadler et al., 2020	C57BL/6 mice	Microbiota-derived SCFAs modulated post-stroke recovery *via* effects on systemic and brain resident immune cells. SCFAs, fermentation products of the gut microbiome, were potent and proregenerative modulators of post-stroke neuronal plasticity at various structural levels. This effect was mediated *via* circulating lymphocytes on microglial activation.	As a link along the gut-brain axis, SCFAs could be a potential therapeutic to improve recovery after ischemic stroke.
Huang J. T. et al., 2021	C57BL/6 mice	Calorie restriction led to better long-term rehabilitation after ischemic stroke in comparison of normal control. Transplantation of gut microbiome from calorie-restriction-treated mice to post-stroke mice was eligible to obtain better long-term rehabilitation of stroke mice.	Calorie restriction conferred improvement effect on long-term rehabilitation of ischemic stroke *via* gut microbiota.
Akhoundzadeh et al., 2018	C57BL/6 mice	Pretreatment with probiotics significantly reduced infarct size by 52% in the mouse MCAO model. Administration of probiotics significantly decreased malondialdehyde content and TNF-α level in the ischemic brain tissue.	Probiotic supplements might be useful in the prevention or attenuation of brain ischemic injury in patients at risk of ischemic stroke.
Sun et al., 2016	C57BL/6 mice	Clostridium butyricum significantly improved neurological deficit, relieved histopathologic change, decreased MDA contents and increased SOD activities in the I/R injury mice. After Clostridium butyricum pretreatment, the expression of Caspase-3 and Bax were significantly decreased, the Bcl-2/Bax ratio was significantly increased, and butyrate contents in the brain were significantly increased.	Clostridium butyricum could exert neuroprotective effects against I/R injury mice through anti-oxidant and anti-apoptotic mechanisms, and reversing decrease of butyrate contents in the brain might be involved in its neuroprotection.
Spychala et al., 2018	C57BL/6 mice	The microbiota was altered after experimental stroke in young mice and resembled the biome of uninjured aged mice. Altering the microbiota in aged to resemble that of young increased survival and improved recovery following MCAO.	The gut microbiota can be modified to positively impact stroke outcomes from age-related diseases.
Wang et al., 2021	C57BL/6 mice	Stroke mice that received gut microbiota from sodium butyrate-treated mice had a smaller cerebral infarct volume than mice that received gut microbiota from NaCl-treated mice. This protection was also associated with improvements in gut barrier function, reduced serum levels of LPS, LPS binding protein, and proinflammatory cytokines, and improvements in the BBB.	The gut microbiota changes of mice aggravated brain injury after ischemic stroke and could be modified by sodium butyrate to afford neuroprotection against stroke injury.
Winek et al., 2016	C57BL/6 mice	When the antibiotic cocktail was stopped 3 days before surgery, microbiota-depleted mice with MCAO had significantly reduced survival compared to MCAO specific pathogen-free and sham-operated microbiota-depleted mice. All microbiota-depleted animals in which antibiotic treatment was terminated developed severe acute colitis. This phenotype was rescued by continuous antibiotic treatment or colonization with specific pathogen-free microbiota before surgery.	Conventional microbiota ensures intestinal protection in the mouse model of experimental stroke and prevents development of acute and severe colitis in microbiota-depleted mice not given antibiotic protection after cerebral ischemia.
Benakis et al., 2020b	C57BL/6 mice	Mice treated with a cocktail of antibiotics displayed a significant reduction of the infarct volume in the acute phase of stroke. The neuroprotective effect was abolished in mice recolonized with a wild-type microbiota. Single antibiotic treatment with either ampicillin or vancomycin, but not neomycin, was sufficient to reduce the infarct volume and improved motor-sensory function 3 days after stroke. This neuroprotective effect was correlated with a specific microbial population rather than the total bacterial density.	Targeted modification of the microbiome associated with specific microbial enzymatic pathways may provide a preventive strategy in patients at high risk for ischemic stroke.
Singh et al., 2016	C57BL/6 mice	Recolonizing germ-free mice with dysbiotic post-stroke microbiota exacerbated lesion volume and functional deficits after experimental stroke compared with the recolonization with a normal control microbiota. In addition, recolonization of mice with a dysbiotic microbiome induced a proinflammatory T-cell polarization in the intestinal immune compartment and in the ischemic brain. Moreover, therapeutic transplantation of fecal microbiota normalized brain lesion-induced dysbiosis and improved stroke outcome.	Acute brain lesions induced dysbiosis of the microbiome and, in turn, changes in the gut microbiota affected neuroinflammatory and functional outcome after brain injury through the brain-gut microbiota-immune axis.
Stanley et al., 2016	Human; C57BL/6 mice	The majority of the microorganisms detected in the patients who developed infections after having a stroke were common commensal bacteria that normally reside in the intestinal tracts. The source of the bacteria forming the microbial community in the lungs of post-stroke mice was the host small intestine.	Stroke promotes the translocation and dissemination of selective strains of bacteria that originated from the host gut microbiota.
Xu et al., 2021	Human; C57BL/6 mice	Enterobacteriaceae enrichment was an independent risk factor for patients with acute ischemic stroke in early recovery. Ischemic stroke induced rapid gut dysbiosis with Enterobacteriaceae blooming. Gut dysbiosis was associated with stroke-induced intestinal ischemia and nitrate production. Enterobacteriaceae exacerbated brain infarction by accelerating systemic inflammation. Inhibiting Enterobacteriaceae overgrowth alleviated brain infarction.	Ischemic stroke rapidly triggers gut microbiome dysbiosis with Enterobacteriaceae overgrowth that in turn exacerbates brain infarction.
Tan et al., 2020	Human	TMAO levels showed no significant changes before and within 24 h of acute ischemic stroke treatment but decreased significantly thereafter. Elevated early TMAO levels were associated with poor outcomes of ischemic stroke patients.	TMAO levels decrease with time since stroke onset. Elevated TMAO levels at an earlier period portended poor stroke outcomes.
Zhang J. et al., 2021	Human	Plasma TMAO levels in patients with ischemic stroke were higher than those in controls. Patients with poor outcomes had significantly higher plasma TMAO levels at admission.	Plasma concentrations of gut microbial TMAO are higher in patients with ischemic stroke and related to poor functional outcomes.
Yamashiro et al., 2017	Human	By investigating the gut microbiota and concentrations of organic acids in ischemic stroke patients and normal individuals, it was found that ischemic stroke was independently associated with increased bacterial counts of Atopobium cluster and Lactobacillus ruminis, and decreased numbers of Lactobacillus sakei subgroup. In addition, ischemic stroke was associated with decreased and increased concentrations of acetic acid and valeric acid, respectively.	Gut dysbiosis in patients with ischemic stroke is associated with host metabolism and inflammation.
Zhu et al., 2020	Human	After controlling for potential confounders, multivariable logistic analysis showed that higher level of plasma TMAO was an independent predictor for cognitive impairment in post-stroke patients.	Increasing plasma level of TMAO may be associated with post-stroke cognitive impairment.
Yin et al., 2015	Human	The gut microbiome of stroke and transient ischemic attack patients was clearly different from that of the asymptomatic group. Stroke and transient ischemic attack patients had more opportunistic pathogens. This dysbiosis was correlated with the severity of the disease. The TMAO level in the stroke and transient ischemic attack patients was significantly lower than that of the asymptomatic group.	Stroke and transient ischemic attack patients showed significant dysbiosis of the gut microbiota, and their blood TMAO levels were decreased.
Li et al., 2019	Human	The gut microbiota of ischemic stroke patients had more short chain fatty acids producer than healthy controls.	Ischemic stroke patients show significant dysbiosis of the gut microbiota with enriched short chain fatty acids producer.

*COX-2, Cyclooxygenase-2; iNOS, inducible Nitric Oxide Synthase; SCFAs, short-chain fatty acids; TNF, tumor necrosis factor; NOS2, Nitric Oxide Synthase 2; IL, interleukin; TMAO, trimethylamine N-Oxide; MCAO, middle cerebral artery occlusion; MDA, malondialdehyde; SOD, superoxide dismutase; I/R, ischemia/reperfusion; Bcl-2, B cell lymphoma-2; Bax, BCL-2-associated protein X; ZO-1, zonula occludens-1; LPS, lipopolysaccharide; BBB, blood-brain barrier; IFN, interferon.*

### Neuroendocrine Pathways

Gut-brain-microbiota axis plays an important role in gut microbiota-related stroke pathophysiology. There are several neural pathways for GBMAx communication, such as spinal and vagal pathways, autonomic nervous system (ANS), enteric nervous system (ENS), and hypothalamic-pituitary-adrenal (HPA) axis (Foster and McVey Neufeld, 2013; Carabotti et al., 2015). The function of gastrointestinal ANS will change after ischemic stroke. The production and release of norepinephrine is increased, and cholinergic activity is decreased, which results in altered intestinal mucin production, inhibiting intestinal activity and increasing intestinal permeability (Caso et al., 2009). This can affect the size and quality of the intestinal mucus layer. As the habitat of most intestinal microbiota, the change of the status of the mucus layer can affect the composition and function of the microbiota. Changes in intestinal permeability induced by stress would lead to the activation of glial cells and mast cells, increased production of interferon, and morphological changes of colonic epithelium. These changes are caused by the expression of reduced tight junction protein 2 and the occlusion of an important component of the intestinal tight junction (Demaude et al., 2006). Increased permeability of the intestinal epithelium will cause bacterial antigens to cross the intestinal epithelium and trigger an immune response, resulting in changes in intestinal flora and systemic effects (Yates et al., 2001). Thus, stroke-induced increases in intestinal permeability can be ameliorated after inhibition of β-adrenergic activity with β -blockers. It can also reduce the risk of bacteria spreading to surrounding organs (Stanley et al., 2016). HPA axis is also an important part of GBMAx and plays a role in enter-brain regulation. HPA axis regulates the body through the interaction of three endocrine glands, including hypothalamus, pituitary and adrenal, which can stimulate the release of steroid hormones such as cortisol under stress. Long-term elevation of serum cortisol can have toxic effects on the nervous system. Serum cortisol levels were found to be associated with stroke severity and post-stroke mortality (Christensen et al., 2004; Barugh et al., 2014). In addition, one study suggested that cortisol levels may be associated with gut microbiota diversity (Keskitalo et al., 2021). Interactions of gut microbiota and HPA axis may explain some severe mental disorders after ischemic stroke (Misiak et al., 2020).

Hormone levels are also thought to play a role in ischemic stroke. Deficiency of estrogen and other ovarian hormones was found to be a risk factor for ischemic stroke in post-menopausal women (Reeves et al., 2008). In animal experiments, different effects of estrogen on the prognosis and recovery of stroke rats were closely related to the age of the rats. Many studies demonstrated that neurological function in young female animals was less affected than in older animals after ischemic stroke. And in the same age groups, female animals showed better post-stroke neurological performance than males (Hall et al., 1991; Alkayed et al., 1998; Manwani et al., 2013). Estrogen can effectively prevent the growth of pathogenic bacteria, promote the growth and reproduction of beneficial bacteria, and maintain a reasonable composition of intestinal microbiota (Chen and Madak-Erdogan, 2016; Baker et al., 2017). There are significant differences in intestinal microbiome and metabolites between young and old women. Akkermansia muciniphila, for example, is a bacterium that can affect energy regulation, metabolism, and cardiovascular function (Everard et al., 2013; Plovier et al., 2017). In healthy mice, levels of Akkermansia muciniphila were found to be lower in female mice than in male mice. But after a stroke, its levels were significantly elevated in male mice (Stanley et al., 2018; Park et al., 2020). Estrogen therapy in stroke patients can reduce lipopolysaccharide (LPS) production and increase short-chain fatty acids (SCFAs) levels, which helps to inhibit inflammatory response and reduce brain tissue damage after ischemic stroke (Blasco-Baque et al., 2012; Wu et al., 2021). Transplanting fecal microbiome from young women undergoing estrogen therapy to older women with stroke effectively improved their functional outcomes (Park et al., 2020). However, estrogen therapy, which has a protective effect in younger women, increased the risk and severity of stroke in post-menopausal women (Wassertheil-Smoller et al., 2003; Selvamani and Sohrabji, 2010a,b). These studies suggested that estrogen levels could regulate intestinal microbiome homeostasis after ischemic stroke and influence stroke outcomes. But more research is needed to explore the relationship between hormones, age, gut microbiota, and ischemic stroke.

### Role of Bacterial Metabolite

Gut microbiota plays an important role in the production and secretion of over 100 metabolites. However, the role of these metabolites in neurological function after ischemic stroke has not been fully studied (Bostanciklioğlu, 2019). SCFAs are the main product that produced by gut microbiota through fermentation of dietary fiber, including acetate, propionate, and butyrate. Recent studies suggested that anaerobic bacteria, such as Firmicutes, produced a great amount of SCFAs by fermenting dietary fiber (Nakamura et al., 2017). SCFAs can also be produced by fermentation of proteins and amino acid, and about 1% of *E. coli* bacteria can produce branched SCFAs (such as isobutyric and isovaleric acids) through this pathway (Smith and Macfarlane, 1997; Louis and Flint, 2017). In addition, acetyl-CoA formed by glycolysis can also be converted to butyric acid by the action of Butyryl-CoA: Acetate-CoA transferase (Duncan et al., 2002). Moreover, SCFAs produced by different species of bacteria also varies. For instance, acetate is metabolized by enteric and acetogenic commensal bacteria (Maa et al., 2010). Propionate is the main metabolite of bacteroides and Firmicutes (Zapolska-Downar and Naruszewicz, 2009). Eubacterium, anaerobe and Faecalis are the main bacteria which produce butyrate (Zapolska-Downar et al., 2004).

Short-chain fatty acids have positive effects on human intestinal function, such as enhancing intestinal motility, reducing inflammatory cell level, and regulating intestinal hormones and neuropeptide levels (Silva et al., 2020). SCFAs are also important for cerebral development and maintenance of normal function of the central nervous system (CNS). They can cross the BBB, and are essential for several processes, such as microglial maturation, intestinal neuron stimulation of ANS, and mucosal serotonin secretion (Braniste et al., 2014; Erny et al., 2015). In addition, SCFAs also play an immunomodulatory role. They can induce T cells to differentiate into effector cells and regulatory cells according to the immune environment (Park et al., 2015).

In the early stage of ischemic stroke, the levels of acetic acid and propionic acid were found to be significantly lower than normal. The concentrations of isobutyric acid and isovaleric acid were increased in the early stage but low in the later stage. Butyric acid and valeric acid were deficient in both early and later stages of ischemic stroke (Chen et al., 2019b). This difference may be related to different metabolic pathways required to produce different SCFAs. Studies have demonstrated that SCFAs have a neuroprotective effect. The lower the concentration of acetic acid, valeric acid, especially butyric acid, in stroke patients, the greater the volume of cerebral infarction and the worse the neurological function score (Chen et al., 2019b). Oral infusion of SCFAs-producing bacteria and inulin reduced neurological deficits and improved post-stroke depression-like behavior in elderly mice (Lee et al., 2020). Butyrate has the function of reducing neurotoxicity, alleviating neuroinflammation, and relieving behavioral disorders. Intestinal butyrate supplementation can improve the level of neurological recovery after brain injury and reduce the volume of cerebral infarction. It was also effective in reducing cerebral edema, lowering blood lipid levels, and reducing the risk of thrombosis (Sharma et al., 2015; Patnala et al., 2017). Butyrate can regulate immune function by inhibiting histone deacetylase (HDAC) and mammalian target of rapamycin (mTOR) signal in circulating leukocytes. Studies have shown that higher concentrations of butyrate in feces or intravenous butyrate solution can enhance the antimicrobial activity of monocytes and macrophages and increase the body’s resistance to pathogens (Chakraborty et al., 2017; Haak et al., 2018).

Trimethylamine N-oxide (TMAO) is a kind of metabolic product of gut microbiota, mainly derived from the dietary nutrients rich in phosphatidylcholine, choline, and L-carnitine. First, Gut microbes metabolize foods such as eggs and beef to produce the intermediate trimethylamine by the activity of trimethylamine (TMA) lyases. In the second step, TMA is oxidized to TMAO by hepatic flavin-containing monooxygenases (Bennett et al., 2013). TMAO can induce atherosclerosis by increasing uptake of cholesterol in macrophages and promoting foam cell formation (Wang et al., 2011), enhance platelet hyperresponsiveness and increases the risk of thrombosis by changing stimulus-dependent calcium signal (Zhu et al., 2016). Higher concentrations of TMAO were found to be associated with an increased risk of cardiovascular events (Wang et al., 2011; Koeth et al., 2014; Tang et al., 2014). In addition, high levels of plasma TMAO have been shown to reduce long-term survival in patients with chronic kidney disease (Tang et al., 2015). Notably, a study of TMAO and cardiovascular disease risk in hemodialysis patients showed a significantly higher risk of death in white patients than in blacks (Shafi et al., 2017). This suggests that the effects of TMAO may differ across racial and ethnic groups.

According to the current study, TMAO can aggravate brain injury after ischemic stroke through a variety of pathophysiological processes. In addition to accelerating atherosclerosis and enhancing thrombogenesis potential, it can also promote vascular inflammation and endothelial dysfunction (Seldin et al., 2016; Boini et al., 2017; Li T. et al., 2017). TMAO also increases oxidative stress, enhances mitochondrial damage, and inhibits mTOR signaling, thereby impairing neural function (Chen et al., 2017; Li D. et al., 2018). TMAO is also a risk factor for hypertension and diabetes, which are linked to ischemic stroke. TMAO levels will rise and then decrease gradually over time after stroke onset. High concentrations of TMAO in patients with early onset are often associated with poor prognosis. Therefore, the measurement of plasma concentrations of gut microbial TMAO in stroke patients is helpful for us to predict the prognosis of patients (Tan et al., 2020; Zhang J. et al., 2019).

### Immunological Mechanisms

As a gathering place of immune cells, gastrointestinal tract affects the growth and development of immune cells and plays an important role in regulating immune response (Li et al., 2019). After ischemic stroke, both local neuroinflammatory responses and peripheral immune responses can be activated (Chamorro et al., 2012). It is found that different kinds of immune cells can aggravate the injury or protect the damaged brain tissue, respectively.

After the occurrence of stroke, activation of cerebral resident immune cells such as microglia, astrocytes, neutrophils and macrophages increase the production of pro-inflammatory cytokines, chemokines, proteases, and adhesive proteins (Iadecola and Anrather, 2011; Yang et al., 2019). The activation of inflammatory cells destroys the integrity of BBB, increases the chemotaxis of inflammatory cytokines in cerebral ischemia area, aggravates the damage of brain tissue. Experimental studies have shown that endotoxins metabolized by microbiota, such as LPS, can exacerbate neuroinflammation either directly or by inducing migration of peripheral immune cells to the brain (Lukiw et al., 2018). And raising LPS levels in stroke mice can promote the production of inflammatory factors like interleukin (IL)-6 and tumor necrosis factor (TNF)-α, affects BBB function, increase the neurological impairment, aggravating cerebral edema and reduce life expectancy of the mice (Dénes et al., 2011). It also led to increased plasma levels of pro-inflammatory cytokines that may promote dysregulation of the gut microbiome (Yamashiro et al., 2017). The dysbiosis of intestinal microflora can further increase the production of peripheral inflammatory cytokines. These cytokines can cross the BBB and exacerbate brain ischemic injury (Liu et al., 2020). Rapid dysregulation of intestinal flora in the first 24 h after stroke can promote cerebral infarction through inflammatory response. By inhibiting the overgrowth of Enterobacteriaceae and other opportunistic pathogens in stroke patients, systemic inflammation and cerebral infarction can be effectively reduced (Xu et al., 2021).

Peripheral immune inflammatory cells are involved in the cerebral immune inflammatory response following ischemic stroke and play an important role in the process of brain injury and tissue repair. The main cells involved in the human immune system are B lymphocytes, T lymphocytes, MHC and effector cells (Flajnik and Kasahara, 2010). In ischemic stroke, impaired BBB promotes T infiltration and interferon (IFN)-γ accumulation (Kleinschnitz et al., 2010; Liesz et al., 2011). In animal stroke models, T cell and B cell counts in Peyer’s patches decreased within 24 h, and activated T lymphocytes migrate from the Peyer patches of the small intestine or from the intestinal lamina propria to the brain within 2–3 days after stroke, where they primarily located in is leptomeninges (Schulte-Herbruggen et al., 2009). T cells can affect the secretion of cytokines IL-17 and IL-23 (Fan et al., 2020), lead to chemokine production and increased infiltration of cytotoxic cells (neutrophils and monocytes) into brain tissues, and then results in neurotoxic effects on ischemic lesions, resulting in increased infarct volume. Experiments have shown that inhibition of T lymphocyte invasion can effectively reduce the infarct size after stroke (Liesz et al., 2011). Conversely, upregulation of T regulates cell (Treg) level or increases IL-10 concentration can inhibit the production of proinflammatory mediators, thereby reducing the volume of cerebral infarction (Wei et al., 2011; Bodhankar et al., 2015). Similarly, regulatory B lymphocytes may also play a protective role in ischemic stroke by regulating anti-inflammatory factors such as IL-10 and transforming growth factor (TGF)-β (Doyle et al., 2015). Increasing B cell concentration in the brain can reduce infarct volume after stroke (Chen et al., 2012).

Intestinal microbiome dysregulation can reduce systemic anti-inflammatory cytokines such as TGF-β and IL-10 (Yan et al., 2009; Benakis et al., 2016). As a bacterial metabolite, SCFAs can act on immune cells by inhibiting histone deacetylase (HDAC) or by acting as a ligand for G-protein-coupled receptors. After stroke, SCFAs also stimulates the production of colonic Treg cells by producing IL-10 cytokines and TGF-β, and expressing Foxp3 and cell surface markers CD4 and CD25, thereby reducing infarct size (Sadler et al., 2020). In addition, monocytes/macrophages in the intestinal tract of stroke patients can be activated by intestinal flora. Intrusions of intestinal monocytes into the brain can be detected during the acute phase of stroke. Therefore, monocytes/macrophages also play a role in microbiome mediated stroke prognosis (Singh et al., 2016).

### Gut Microbiota-Related Complications Following Ischemic Stroke

Post-stroke infection is an important factor causing worsen outcomes of stroke patients, and more than one-third of patient’s condition and treatment are complicated by post-stroke infection complications (Emsley and Hopkins, 2008). Increased susceptibility to infection after ischemic stroke is associated with activation of feedback activity between the CNS and peripheral immune organs (Chamorro et al., 2012). And according to current studies, the increased permeability and dysfunction of the intestinal barrier after stroke can cause bacterial migration and spread of the intestinal microbiome, which may be one of the mechanisms of post-stroke infection (Figure 1; Stanley et al., 2016).

After ischemic stroke, the sympathetic nervous system is activated, the intestinal permeability is increased, the intestinal barrier is damaged, and the antibacterial function of the body is reduced. These changes promote the transfer of bacteria to extra-intestinal organs, blood or lymph, participates in local and systemic immunity, and may lead to organ infections and even sepsis (Hagiwara et al., 2014). The β-adrenergic signaling pathway may play an important role (Wong et al., 2011). When β-adrenergic receptors are blocked, the integrity of the intestinal barrier can be inhibited. For example, feeding stroke mice with propranolol can’t completely avoid the occurrence of infection, but can obviously reduce the serious situation of systemic tissue infection after ischemic stroke (Stanley et al., 2016).

In an mice experimental study, germ-free mice were modeled by two methods. The results showed that the intestinal microbiota of mice with post-filament middle cerebral artery occlusion model (fMCAo) had significantly imbalance and the diversity was decreased while those in mice with permanent distal middle cerebral artery occlusion model (cMCAo) had relatively little influence. This suggests that the changes in microbiome are secondary to the stroke and the degree of disturbance is affected by the severity of stroke (Singh et al., 2016).

In turn, gut microbiota disturbance may be one of the important causes of enterogenic infection, sepsis even multiple-organ dysfunction syndromes (MODS) (Lyons et al., 2016; Haak and Wiersinga, 2017). After stroke, the destruction of the integrity of the intestinal barrier provides conditions for bacterial migration. Although in healthy individuals a variety of gut microbiomes are also found in blood and lung tissue (Potgieter et al., 2015; Dickson et al., 2016; Li Q. et al., 2018). However, due to the destruction of the integrity of the intestinal barrier, pathologic translocation of intestinal bacteria in stroke patients increases, leading to an increase in the incidence of post-stroke infection (Tascilar et al., 2010).

Bacterial pneumonia is the most common complication of ischemic stroke patients and one of the early nosocomial infections (Langhorne et al., 2000; Hannawi et al., 2013). Blood or sputum culture samples taken from patients with stroke complicated with pneumonia are often absent of the common pathogens that cause pneumonia, or have much less than those found in patients with common pneumonia (Marik, 2001). In one study, prophylaxis with antibiotics in ischemic stroke patients did not reduce the incidence of pneumonia or death compared with untreated patients (Kalra et al., 2015). In contrast, evidence from several studies suggests that post-stroke pneumonia is associated with the transmission of certain bacteria from the patient’s gut microbiota. These bacteria, when translocated, become pathogenic strains (Stanley et al., 2016). This suggests that endogenous factors play a more important role in the onset of post-stroke pneumonia than exogenous infection. Changes in intestinal permeability after ischemic stroke can induce bacterial migration and infection. In addition to direct transmission through the small intestine after, gut bacteria can also travel through the portal vein to the liver, where they can spread indirectly to the lungs after filtering through the blood.

Neuropsychiatric disorders are also common complications of stroke, includes depression, anxiety, mania, dementia, and cognitive impairment (Hackett et al., 2014; Hackett and Pickles, 2014). About one-third of patients experience cognitive impairment within a year of a stroke (Li X. et al., 2017). Gut microbiota dysbiosis can be found in many neurological disorders such as Alzheimer’s disease and depression (Cho et al., 2021). The high-abundance Prevotella group expressed more negative emotions and reduced hippocampal functional activation than the group with higher levels of bacteroides (Tillisch et al., 2017). In addition, bacterial metabolites have been linked to cognitive function. Ischemic stroke patients who detect higher levels of TMAO experience more severe cognitive impairment (Zhu et al., 2020). SCFAs producing bacteria (such as Lachnospiraceae and Ruminococcus) were significantly reduced in patients with amnestic cognitive impairment (Liu et al., 2021). Another study found similar results. The abundance of Lachnospiraceae, Clostridiaceae, and Ruminococcus was reduced in the high-risk group compared with the low-risk group (Huang Q. et al., 2021). Gut microbiota may aggravate neuropsychiatric symptoms by common pathogenesis, like neuroinflammatory response. These results suggest that it is feasible to predict the occurrence of cognitive impairment after ischemic stroke by intestinal flora.

### Gut Microbiota With Other Types of Stroke

Current studies on the gut-brain axis mainly focus on patients with ischemic stroke. Compared with ischemic stroke, there are fewer clinical and experimental studies on the association between hemorrhagic stroke and intestinal flora. The occurrence probability of intracranial hemorrhage (ICH) and subarachnoid hemorrhage (SAH) are less than that of cerebral infarction, but the mortality and disability rate of ICH and SAH are not low. Although the pathogenesis and clinical manifestations of the two types of stroke patients are different, similar results of microbiome disruption were found between the two stroke patients. Some studies have even shown that the stability disruptions of gut microbiota in patients with hemorrhagic stroke or high NIHSS scores are more severe than those with ischemic stroke and TIA (Zeng et al., 2019; Haak et al., 2021). Like ischemic stroke, some mechanisms of action are also at work in ICH patients. According to a case-control study of hypertension patients in China, intestinal bacterial metabolite TMAO levels are strongly associated with stroke. The association between TMAO and hemorrhagic stroke was significantly higher than that of ischemic stroke (Nie et al., 2018). TMAO has also been shown to be closely associated with the prognosis of ICH patients (Zhai et al., 2021). Inflammation has also been found to play an important role in the brain-gut axis in patients with intracerebral hemorrhage. An animal study has demonstrated that dysregulation of gut flora is associated with dysregulation of pro-inflammatory T cell differentiation in mice after intracerebral hemorrhage, which exacerbates neuroinflammatory responses and causes secondary damage to brain tissue. Neuroinflammation was reduced in ICH mice after intestinal transplantation with fecal gut microbiota from healthy mice (Yu et al., 2021). Intestinal disruption also occurred after ICH. Persistent ileal mucosal injury and increased intestinal permeability were observed in ICH mice. This permeability reached its highest level on the 7th day after intracerebral hemorrhage. Intestinal disruption also occurred after ICH. Persistent ileal mucosal injury and increased intestinal permeability were observed in ICH mice. This permeability reached its highest level on day 7 of intracerebral hemorrhage. Intestinal bacteria can enter the blood circulation through the broken intestinal mechanical barrier, and lead to systemic inflammation, especially pneumonia (Zhang H. et al., 2021).

As the most common cause of SAH, intracranial aneurysms have a prevalence of about 3% in the population and are associated with 80–85% of non-traumatic SAH (Kassell et al., 1990; Vlak et al., 2011). The current study reveals a partial link between intracranial aneurysms and gut microbiota. Destruction of the gut microbiota by antibiotics can reduce the incidence of intracranial aneurysms in mice (Shikata et al., 2019). And in another study, intracranial aneurysm formation can be induced in normal mice after transplantation of feces from patients with an intracranial aneurysm. Further studies revealed a relationship between aneurysms and the abundance of H. hathewayi in the gut (Li H. et al., 2020). This is a group of anaerobic bacteria that can maintain stable levels of serum taurine, which reduces the risk of aneurysm formation and rupture by inhibiting systemic inflammation. Meanwhile, artificial taurine supplementation also reversed the progression of intracranial aneurysms (Li H. et al., 2020). On the other hand, intestinal microbiota can also play an important role in the rupture of aneurysms. Compare the gut microbiota of patients with ruptured aneurysms (RA) and unruptured aneurysms (URA), researchers found that the genus Campylobacter and C. ureolyticus were significantly increased in the RA group patients (Kawabata et al., 2022). This may be related to the more intense inflammatory response and the remodeling or destruction of blood vessel walls induced by Campylobacter (Nilsson et al., 2018; Kushamae et al., 2020).

Cavernous hemangioma (CA) is also a kind of common vascular disorder will cause cerebral hemorrhage. More abundance Gram-negative bacteria O. splanchnicus and lower levels of gram-positive bacteria F. prausnitzii and B. adolescentis can be found in CA patients, compared with the non-CA patients. However, the most valuable combination of bacteria for diagnosing disease and assessing its severity was not found in this experiment. This indicates that the influence of bacteria on CA is not independent but may play a role together with other factors.

### A Promising Biomarker for Predicting Stroke Outcomes

Currently, it has been proved that there is a correlation between intestinal microbiota dysbiosis after stroke and the incidence and progression of stroke. Therefore, it is feasible to predict the disease recovery of patients by indicators related to intestinal microbiome. By comparing samples from stroke patients and control group, several studies got the similar conclusions that stroke patients had a reduction in Firmicutes and Bacteroidetes, while the abundance of Proteobacteria was increased. And the difference of composition ratio in the microbiome was correlated with the severity of the disease. The abundance of TMA-producing bacteria was significantly higher, and the levels of intestinal butyrate-producing bacteria decreased in severe patients compared with mild patients. And the metabolites like TMA or butyrate of these bacterias also had a similar situation (Gu et al., 2021; Haak et al., 2021; Xia et al., 2021).

By examining the difference in intestinal microbiota distribution between acute ischemic stroke patients and healthy participants, a study established a Stroke Dysbiosis Index (SDI), as an independent predictor of severe disease (NIHSS > 8) and poor prognosis (MRS > 2) (Xia et al., 2019). Increased abundance of Enterobacteriaceae and Parabacteroides have a correlation with a higher SDI, while the abundance of fecalibacterium, Clostridiaceae, and Lachnospira decreased. In addition, animal studies have shown that mice that received fecal transplants from patients with a high SDI index experienced severe brain damage, increased levels of IL-17 and T cells, and a significantly higher risk of stroke than mice that received normal fecal transplants.

### Treatment and Management Strategies Targeting Gut Microbiota for Ischemic Stroke

#### Dietary Interventions

**FIGURE 2:**
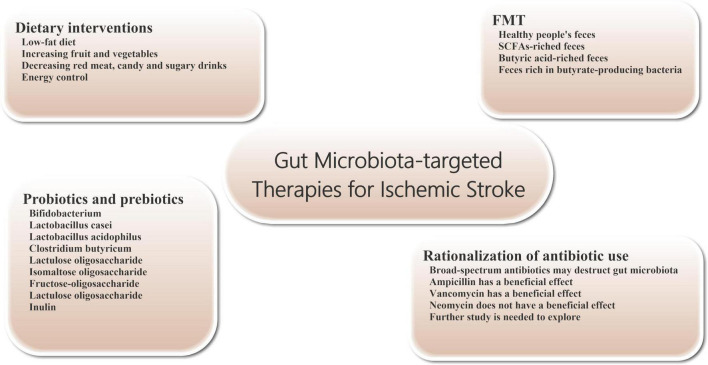
Gut microbiota-targeted treatments and managements for ischemic stroke. Gut microbiota-targeted treatments and managements can be considered for patients with ischemic stroke, including dietary interventions, probiotics and prebiotics supplementation, FMT, and rationalization of antibiotic use. FMT, fecal microbiome transplantation.

Dietary regulation is an important measure to improve the prognosis of stroke (Figure 2). At the same time, diet, smoking cessation and blood pressure control are also three important interventions to prevent stroke (Hackam and Spence, 2007). A low-fat diet is recommended to reduce the risk of cerebrovascular disease. Many of the guidelines recommend a diet that reduces saturated fat and cholesterol and increases fruit and vegetables. Specifically, it includes vegetables, grains, poultry, fish and nuts, and cuts out red meat, candy and sugary drinks (Juraschek et al., 2017). While meat from different animals has roughly the same amount of cholesterol, red meat is higher in saturated fat and has about four times as much carnitine as chicken and fish. Carnitine and choline can be converted to TMAO by intestinal bacteria, affecting stroke outcomes. Therefore, stroke patients and high-risk patients should avoid foods such as red meat and egg yolks.

In addition, increasing the consumption of fruits and vegetables can increase fiber intake, which can increase the level of SCFAs production. For example, resistant starches (such as whole grains and legumes) and fructo-oligosaccharides (such as bananas, Onions and asparagus), as metabolic food sources for butyric acid producing bacteria, can increase butyric acid production in the gut (Le Blay et al., 1999).

Energy control is an effective way to promote good health and reshape the intestinal symbiotic microbiome. Some studies suggest that energy restriction to 60–70% of the recommended intake is protective against ischemic stroke (Mitchell et al., 2019). The protective effect of energy control on brain injury after stroke may be realized by promoting glycogen metabolism and adiponectin expression (Ciobanu et al., 2017; Zhang J. et al., 2019). And long-term energy control resulted in significant changes in the composition of intestinal flora in mice experiments, especially the enrichment of bifidobacterial (Huang J. T. et al., 2021).

#### Probiotics and Prebiotics

Probiotics are a group of living gut microorganisms that are widely believed to be beneficial to the host. Probiotics can affect brain function by altering brain neurochemistry. Current studies have shown that probiotics supplementation can effectively reduce or prevent brain tissue damage in stroke patients. Probiotics may protect tissue from damage by reducing the production of oxygen free radicals and inflammatory cytokines. For example, probiotics can inhibit the production of TNF-α *in vivo*, promote the generation of anti-inflammatory cytokines, and improve the activity of antioxidant enzymes (Luo et al., 2014; Abhari et al., 2016). The severity of brain tissue damage in mice after focal cerebral ischemia was significantly reduced by 2 weeks of daily intake of probiotics such as bifidobacterium, Lactobacillus casei, Lactobacillus bulgaricus and Lactobacillus acidophilus (Akhoundzadeh et al., 2018). Pretreatment with Clostridium butyricum can effectively inhibit apoptosis and enhance antioxidant enzyme activity in rat cerebral ischemia model, thereby improving prognosis (Sun et al., 2016). In addition, regular consumption of lactobacillus probiotics can also alter the expression of brain-derived neurotrophic factor (BDNF) receptors and increase BDNF levels in the brain. There is evidence that elevated levels of BDNF in the brain have a protective effect on ischemic stroke (Bercik et al., 2011; Liang et al., 2015).

Prebiotics are oligosaccharides with no biological activity, such as lactulose oligosaccharide, isomaltose oligosaccharide, fructose-oligosaccharide, lactulose oligosaccharide and inulin, which can stimulate the growth and reproduction of beneficial bacteria in the intestine without being digested by intestinal metabolism. After entering the intestinal tract (mainly the lower digestive tract or colon), prebiotics can be hydrolyzed and used as nutrients by the beneficial bacteria in the intestinal tract, such as bifidobacterium, and promoting the reproduction and growth of these bacteria. In addition, prebiotics can also affect the production of SCFAs and regulate the production of mucin, thus enhancing the phagocytosis of macrophages (Markowiak and Śliżewska, 2017). In one study, prebiotics effectively reduced the incidence and severity of pneumonia during hospitalization in critically ill patients. Therefore, we believe that the use of prebiotics can play a certain role in alleviating ischemic stroke patients’ condition and the onset of infectious complications (Barraud et al., 2013).

#### Fecal Microbiome Transplantation

The transfer of the entire microbiome from the stool of a healthy donor to the patient’s gastrointestinal tract is known as fecal microbiome transplantation (FMT). The technique is already being used to treat patients with severe infections, such as refractory bronchiolitis and pseudomembranous colitis (Eiseman et al., 1958; van Nood et al., 2013). In addition, FMT intervention can also relieve symptoms in patients with Parkinson’s disease and reduce autism in children with autism disorder (Aroniadis and Brandt, 2013; Kang et al., 2017). However, because the gut microbiome also has the potential to cause disease, it is important to select suitable healthy people as FMT donors. Transplantation SCFAs-riched feces (particularly butyric acid) can regulate the composition of intestinal microbes, increase lactobacillus species and enhance microbial activity, maintain intestinal wall integrity and reduce intestinal wall permeability, thereby reducing intestinal leakage in patients with ischemic stroke. These positive effects are beneficial to maintain the integrity of BBB and improve the functional status of brain tissue in ischemic stroke patients (Singh et al., 2016; Chen et al., 2019a,b). For example, transplanting gut microbiota from young mice can improve stroke outcomes in older mice (Spychala et al., 2018). Transplantation of feces rich in butyrate-producing bacteria has also been shown to reduce ischemic stroke injury in diabetic mice (Wang et al., 2021).

#### Rationalization of Antibiotic Use

In clinical work, about 30% of patients with stroke will have bacterial infection within 1 week of onset (Westendorp et al., 2011), so a significant number of patients receive prophylactic anti-infective therapy with antibiotics, which often include a combination of broad-spectrum antibacterial drugs. However, to date, there is no clear evidence that prophylactic use of antibiotics after stroke benefits patient outcomes. Compared with standard treatment regimen, prophylactic use of antibiotics in stroke patients did not improve the long-term neurological status or mortality and had no significant effects on the incidence of post-stroke complications such as pneumonia. In some patients, intestinal microbiota damage can promote immune suppression, which increases the probability of facultative or mandatory bacterial re-invasion and increases the risk of infection (especially pneumonia) (Kalra et al., 2015; Westendorp et al., 2015). Further studies have shown that extensive destruction of the gut microbiome by untargeted use of broad-spectrum antibiotics after ischemic stroke can worsen stroke outcomes (Benakis et al., 2016; Winek et al., 2016). Studies have shown neuroprotective effects on brain tissue in stroke mice treated with ampicillin or vancomycin. A similar neuroprotective effect was not observed with neomycin (Benakis et al., 2020a,b). These different effects may be related to changes in the composition of intestinal flora. Therefore, further work is needed to explore whether specific antibiotics can have a beneficial effect on the prognosis of patients with ischemic stroke.

## Conclusion and Perspective

As the most common type of stroke, treatment options for ischemic stroke remain limited despite extensive research. New insights have highlighted the role of gut microbiota in the pathophysiology of ischemic stroke. Ischemic stroke could cause gut microbiota dysbiosis as well as translocation and dissemination of gut microbiota-derived selective strains of bacteria. In turn, changes in gut microbiota affect ischemic stroke-induced brain injury and determine stroke outcomes through multiple mechanisms, including neuroendocrine pathways, bacterial metabolite, and immune response. Gut microbiota dysbiosis may also contribute to some stroke complications such as pneumonia, sepsis, and neuropsychiatric disorders. Some gut microbiota-targeted therapies have shown potential in the treatment and management of ischemic stroke, including dietary interventions, probiotics supplementation, FMT, and rationalization of antibiotic use. Gut microbiota is expected to provide new perspectives for ischemic stroke treatment. However, the efficacy and safety of this treatment strategy for ischemic stroke have not been verified in large scale clinical trials. In addition, it must be recognized that gut microbiota dysbiosis is only one component of the multifactorial brain injury mechanisms of ischemic stroke. Further studies are necessary to broaden our knowledge of the role of gut microbiota in the pathogenesis of ischemic stroke and to facilitate the development of novel therapeutic strategies for ischemic stroke.

## Author Contributions

ZB wrote the manuscript. ZZ directed the writing of the manuscript, revised the manuscript, and made the figures and the table. GZ and AZ checked the manuscript. AS proposed the idea. FZ supervised the writing of the manuscript. All authors approved the submitted version.

## Conflict of Interest

The authors declare that the research was conducted in the absence of any commercial or financial relationships that could be construed as a potential conflict of interest.

## Publisher’s Note

All claims expressed in this article are solely those of the authors and do not necessarily represent those of their affiliated organizations, or those of the publisher, the editors and the reviewers. Any product that may be evaluated in this article, or claim that may be made by its manufacturer, is not guaranteed or endorsed by the publisher.
